# Gene signatures ESC, MYC and ERG-fusion are early markers of a potentially dangerous subtype of prostate cancer

**DOI:** 10.1186/1755-8794-7-50

**Published:** 2014-08-12

**Authors:** Morten Beck Rye, Helena Bertilsson, Finn Drabløs, Anders Angelsen, Tone F Bathen, May-Britt Tessem

**Affiliations:** 1 Department of Cancer Research and Molecular Medicine, Norwegian University of Science and Technology (NTNU), P.O. Box 8905, N-7491 Trondheim, Norway; 2 St. Olavs Hospital, Trondheim University Hospital, Trondheim, Norway; 3 Department of Urology, St. Olavs Hospital, Trondheim University Hospital, Trondheim, Norway; 4 MI Lab, Department of Circulation and Medical Imaging, Norwegian University of Science and Technology (NTNU), Trondheim, Norway

**Keywords:** Prostate cancer, Gleason, Subtypes, Microarray, GSEA

## Abstract

**Background:**

Good prognostic tools for predicting disease progression in early stage prostate cancer (PCa) are still missing. Detection of molecular subtypes, for instance by using microarray gene technology, can give new prognostic information which can assist personalized treatment planning. The detection of new subtypes with validation across additional and larger patient cohorts is important for bringing a potential prognostic tool into the clinic.

**Methods:**

We used fresh frozen prostatectomy tissue of high molecular quality to further explore four molecular subtype signatures of PCa based on Gene Set Enrichment Analysis (GSEA) of 15 selected gene sets published in a previous study. For this analysis we used a statistical test of dependent correlations to compare reference signatures to signatures in new normal and PCa samples, and also explore signatures within and between sample subgroups in the new samples.

**Results:**

An important finding was the consistent signatures observed for samples from the same patient independent of Gleason score. This proves that the signatures are robust and can surpass a normally high tumor heterogeneity within each patient. Our data did not distinguish between four different subtypes of PCa as previously published, but rather highlighted two groups of samples which could be related to good and poor prognosis based on survival data from the previous study.The poor prognosis group highlighted a set of samples characterized by enrichment of ESC, ERG-fusion and MYC + rich signatures in patients diagnosed with low Gleason score,. The other group consisted of PCa samples showing good prognosis as well as normal samples. Accounting for sample composition (the amount of benign structures such as stroma and epithelial cells in addition to the cancer component) was important to improve subtype assignments and should also be considered in future studies.

**Conclusion:**

Our study validates a previous molecular subtyping of PCa in a new patient cohort, and identifies a subgroup of PCa samples highly interesting for detecting high risk PCa at an early stage. The importance of taking sample tissue composition into account when assigning subtype is emphasized.

## Background

Identification of molecular alterations in prostate cancer (PCa) using gene expression measurements enables subtyping of tumors which can identify molecular risk profiles and therefore improve clinical outcome. In addition to relevant clinical parameters such as prostate specific antigen (PSA) and Gleason score, knowledge about relevant prognostic gene expression subtypes could help in improving personalized treatment strategies both in postoperative follow-up and in recruitment to active surveillance.

Gene expression based subtypes of breast cancer associated with significant differences in prognosis were established more than 10 years ago [1]. Attempts have been made to define subtypes in PCa in a similar manner [2,3], but no robust subtypes have currently been established. One reason may be the highly heterogeneous tissue composition in PCa tissue samples, where the varying amounts of cancer and normal tissue (benign epithelial and stromal cells) may have large impact on gene expression levels in each sample [4]. In addition to compositional variations, PCa is a highly heterogeneous disease manifested by a large variability in molecular tumor characteristics between different patients [5]. Also the degree of genetic heterogeneity in multiple tumor nodes within the same patient due to polyclonal composition remains an open question [6,7]. The identification of molecular markers which characterize PCa at the level of individual genes has so far been challenging [8,9]. It has thus been suggested that the molecular variation underlying the observed differences of PCa, as well as other cancers, does not manifest itself at the level of individual gene expression, but rather at the level of biological pathways and modules of functionally related genes, often represented as gene sets [10-12].

Markert et al. [3] recently investigated the heterogeneity in PCa by clustering sample signatures based on Gene Set Enrichment Analysis (GSEA) from 15 pre-selected gene sets to identify subtypes of PCa. The gene sets for subtype assessment were all chosen due to their association with cancer in general and PCa in particular (see below)**.** Using the signature, Markert et al. [3] identified five and four subtypes, respectively, in two different patient cohorts; a watchful-waiting cohort from Sweden [8] and a cohort subjected to radical prostatectomy from Memorial Sloan-Kettering Cancer center [13]. They related the different subtypes to patient survival, and identified two subtypes characterized by high lethality and bad prognosis, as well as three (two) less dangerous subtypes characterized by good prognosis. One subtype was particularly aggressive showing high scores for gene sets related to embryonic stem cell (ESC) characteristics, proliferation, and PTEN mutation. A gene set related to P53 mutation was also enriched in this bad prognosis subtype in the watchful waiting cohort, but not in the other cohort. Another subtype was moderately aggressive and characterized by high scores for a gene set related to TMPRSS2-ERG fusion, a gene fusion regularly found in PCa [14]. Markert et al. [3] concluded that their five (four) subtypes could have clinical implications in predicting adverse outcome for PCa patients with low Gleason score. Today, low-risk PCa patients (PSA <10-15 ng/ml, Gleason score <7 and organ-confined cancer [15]) should be offered deferred treatment (active surveillance) to minimize the risk of overtreatment. However, due to the lack of good prognostic models, a number of these patients will harbor a more aggressive disease than expected and experience poor survival if not referred to radical treatment. If the results by Markert et al. [3] can be reproduced and introduced to a clinical setting, decision making for inclusion into active surveillance programs and postoperative follow-up for patients with a poor genetic signature can be facilitated.

Using fresh frozen radical prostatectomy prostate tissue material [16], we have previously analyzed gene expression profiles in 156 samples (116 PCa and 40 normal) from 41 patients using microarray technology [17]. All analyzed tissue samples were proven to be of high molecular quality, and thorough histopathological evaluation to characterize the composition of the relative amounts of cancer and normal tissue was performed. Several samples were taken from the same patient, enabling the possibility to evaluate the intra-patient molecular variability. In the current study, we used our previously generated gene expression profiles to validate the PCa subtypes introduced by Markert et al. [3]. Reasons for mis-classifications and identification of molecular similarities in samples from PCa patients with poor prognosis and low Gleason score were especially considered. Furthermore, the significance of sample composition (cancer and normal tissue) on subtype classification was evaluated and the persistency of the subtype classification in samples with different Gleason score from the same patients was assessed.

### Gene signatures in prostate cancer

Tumors originating from a variety of tissues and organs have shown properties characteristic of embryonic stem cells (ESCs). These ESC characteristics are often associated with aggressiveness and poor prognosis, especially in epithelial cancers like breast cancer [18,19]. The special properties of stem cells, such as proliferation, differentiation and self-renewal express a more malignant phenotype. ESC characteristics are also typical for induced pluripotent stem cells (iPSCs), where transcription factors characteristic for ESCs are used to introduce ESC-like properties to normal cells [20]. An upregulation of ESC genes are generally accompanied by a reduced expression of genes regulated by the polycomb repressive complex (PRC). PRC regulation is particularly important in ESCs, where polycomb repress so-called bivalent genes which are either activated or repressed depending on the further path of differentiation [21]. Early dysregulation by the PRC complex in ESCs has been shown to result in a phenotype where PRC-regulated genes are generally repressed, and this is characteristic for poorly differentiated cancers [22].

Another characteristic feature of aggressive cancers is the activation of the MYC oncogene. MYC is a constitutively expressed transcription factor with many targets in different contexts, and it was recently suggested that its main function is to reinforce transcription of already expressed genes [23,24]. A specific MYC module active in ESCs was recently shown to be particularly important for cancer development [25]. ESC, PRC and MYC are all properties shared by tumors from several types of cancer. The aberrant fusion between androgen regulated genes, in particular TMRPSS2, and members of the ETS family of transcription factors (most commonly ERG) is specific to PCa. TMRPSS2-ERG fusion is present in about 50% of all PCa tumors [26]. Prostate tumors positive for ERG-fusion are characterized by an upregulation of targets for the transcription factor ERG, which in turn may lead to upregulation of Polycomb genes, thus linking ERG-fusion to Polycomb repression [27].

Mutations in the tumor suppressor genes P53 and PTEN, together with the oncogene RAS are frequently found in tumors. P53 and/or PTEN mutations are both common in PCa, and have recently been shown to increase proliferation [28]. However, the role of RAS mutation in PCa is less clear [29,30]. Epithelial to Mesenchymal Transition (EMT) is a general characteristic for high grade metastatic tumors resulting in increased cell motility and invasiveness [31]. This aggressive tumor property is generally driven by the transformation of epithelial tumor cells into mesenchymal tumor cells. Inflammation, on the other hand, affects tumors at all stages of development, from initiation through cancer progression and metastasis, and is mainly regulated through the production of tumor promoting cytokines, both in the tumor and in the tumor microenvironment [32]. In the prostate, inflammation manifests itself as Proliferative Inflammatory Atrophy (PIA) lesion which may both precede tumor formation and infiltrate the tumor as it develops [33]. Finally, normal and tumor neuroendocrine cells in the prostate have been shown to participate in the oncogenic processes in PCa progression, and ultimately to the transformation of the tumor to an androgen independent phenotype [34].

## Methods

### Patients and PCa samples

All patients scheduled for radical prostatectomy at St. Olav Hospital, Trondheim University Hospital are invited to donate a 2 mm transversal tissue slice which is collected and stored for relevant research projects in the Regional Research Biobank of Central Norway. All samples for this study were extracted in frozen conditions by a highly specialized harvesting method which is presented and published by our research group [16]. By cutting 4 μm cryosections from the extracted cylinder tissue samples (mean weigh: 12.7 mg, 2 mm thick), histopathology, including estimation of amount of stroma, benign epithelium, cancer and Gleason grade was confirmed before any of the analyses were initiated. Normal samples were taken as far away from the tumor as possible on each tissue slice. The selected samples were used for RNA extraction and microarray analyses, obtaining transcriptomic data, described in detail in a previous publication [17]. The study is approved by the Regional Committee for Medical and Health Research Ethics (REC), Central Norway, and the Data Inspectorate of Norway. A set of microarray measurements on PCa tissue samples used for validation was downloaded from GEO accession GSE8218 [35,36]. Details of this datasets and validation results are given in Additional file 1: Text S1.

### Microarray analysis

A detailed description of the steps used for microarray analysis to obtain gene expression levels are given in a previous publication [17]. The microarray data are publicly available in Array Express with accession number E-MTAB-1041. Metadata consisting of sample composition (cancer and healthy stromal/epithelial cells) and patient number for each sample is found in Additional file 2.

### Gene set enrichment analysis

We downloaded 21 gene sets related to prostate cancer from Markert et al. [3]. A sufficient set of genes for all signatures were present in our microarray data (Additional file 1: Table S2, and Additional file 3), and Gene Set Enrichment Analysis (GSEA) [3,37] was performed for each gene set in each of the 156 samples. Prior to GSEA, each gene was mean-centered across all samples. In each sample, genes were then sorted in descending order according to their normalized and mean-centered microarray expression values. Positive and negative GSEA scores were then calculated for each gene set in each sample by the following formulas:

$$\mathrm{Scor}e_{\mathrm{GS}}^{+}\left(\mathrm{sample}\right)=\mathrm{ma}x_{1\leq k\leq N}\left(\sum_{1\leq j\leq k}d_{\mathrm{GS}}\left(j\right)\right)$$

$$\mathrm{Scor}e_{\mathrm{GS}}^{-}\left(\mathrm{sample}\right)=\mathrm{mi}n_{1\leq k\leq N}\left(\sum_{1\leq j\leq k}d_{\mathrm{GS}}\left(j\right)\right)$$

Here

$$d_{\mathrm{GS}}\left(j\right)=\;\sqrt{\frac{\left(N-S\right)}{S}}$$

if gene *j* is in gene set GS, and

$$d_{\mathrm{GS}}\left(j\right)=\;-\sqrt{\frac{S}{\left(N-S\right)}}$$

if gene *j* is not in signature GS, where N is the total number of genes, and S is the number of genes in the gene set. This produces one positive and one negative score for each gene set in each sample. A total score is calculated by subtracting the negative from the positive score, resulting in one total gene set score for each of the 21 gene sets in each sample. The number of gene set scores was then reduced to 15 by averaging over scores from related gene sets (Additional file 1: Table S2). For normalization of gene set scores we tested three different strategies: i) Normalization to zero-to-one scale across each sample, ii) normalization to zero-to-one scale across each gene set, and iii): normalization to p-values by repeated sampling of random gene sets [3]. The two former normalizations showed similar performance, and both performed better than the p-value normalization. We chose to use normalization for each sample, because this would mostly resemble a clinical setting where standard gene sets are tested on new patients.

### Correlation of sample signatures to previously identified subtypes of prostate cancer

**Figure 1:**
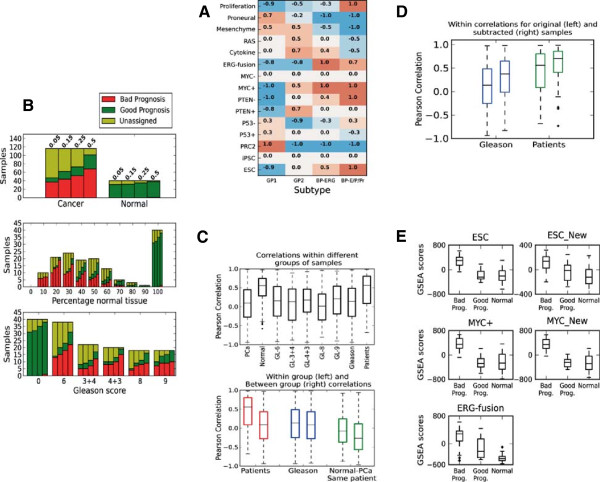
**Gene signatures are consistent within samples from the same patient, but are also affected by tissue composition within each sample. A)** Numerical assessment of signatures for four subtypes of prostate cancer based on the heatmap in Figure four(B) in Markert et al. [3]. **B)** Assignment of PCa samples to subtypes with poor prognosis and subtypes with bad prognosis. The bars show number of samples assigned to the good and bad prognosis subtypes at different p-value thresholds (0.05, 0.15, 0.25 and 0.5 from left to right). **B-Top)** At a p-value threshold of 0.05 (0.25) 37 (52) out of 116 PCa samples are assigned with bad prognosis, and 10 (21) with good prognosis, while 31 (35) out of 40 normal samples are assigned with good prognosis. **B-Middle)** Sample assignment is strongly dependent on the relative amounts of cancer and normal tissue in each sample. **B-Bottom)** PCa sample assignment did not depend on Gleason score, and samples are equally likely to be assigned with poor and good prognosis regardless of Gleason score. **C-Top)** Signature correlations between PCa samples from the same patients are better than signature correlations between samples with the same Gleason scores. This was also the case when samples from the same patient had different Gleason scores. **C-Bottom)** Signature correlations within and between various sample groups. Normal samples are more similar to PCa samples when taken from the same patient (*Normal-PCa – Same patient)* . **D)** Subtracting the average normal signature improves sample similarity within patient and Glesaon grooups. **E)** Bad prognosis samples show elevated scores in MYC, ESC and ERG-fusion gene sets. The three samples sets are: i: *Bad prog*) 54 PCa samples bad prognosis (cluster 1, Figure 2) ii: *Good prog*) 21 PCa samples initially assigned with good prognosis (p<0.25, Table 1A) and iii: *Normal)* 40 normal samples.

We visually translated the heatmap from Markert et al. (Figure four B) based on samples from a radical prostatectomy cohort from Memorial Sloan-Kettering Cancer center to numeric values (Figure 1A). The four subtypes are categorized into two subtypes with bad prognosis; one characterized by enrichment for PTEN-, ESC and Proliferation signatures (BP-E/P/Pr), and one by specific enrichment of the ERG-fusion gene set (BP-ERG), and two subtypes with good prognosis (GP1 and GP2). The scores were defined by a visual inspection of the heatmap, assigning values between -1 and 1 manually according to the color code. The four resulting subtype signatures were correlated to the signatures calculated from our 156 samples using Pearson correlation. A sample was defined as significantly belonging to one of the four subtypes, if the signature correlation to this subtype was significantly better compared to all other subtypes. For this analysis we implemented a statistical test of dependent correlations [38] for various p-value thresholds. In the second test, we investigated whether a sample was significantly better correlated to one of the two subtypes showing poor prognosis (subtype 1 – BP-E/P/Pr and 2 – BP-ERG in Markert et al.) than to the two subtypes showing good prognosis (subtype 3 – GP1 and 4 – GP2 in Markert et al.). To be assigned to poor prognosis subtype, the correlation to either subtype BP-E/P/Pr or BP-ERG must be significantly better than to both subtype GP1 and GP2, but not necessarily significantly different between subtypes BP-E/P/Pr and BP-ERG. Accordingly, to the poor prognosis subtype, the correlation to either subtype GP1 or GP2 must be significantly better than to both subtype BP-E/P/Pr and BP-ERG, but not necessarily between subtypes GP1 and GP2. For this test we used the test of dependent correlations for different p-value thresholds.

### Calculation of within and between group signature correlations

Within and between correlations were calculated for each considered group. Within correlations of cancer and normal samples are all possible correlations within all cancer and normal samples respectively. For Gleason score, within correlations are all correlations within cancer samples classified with the same Gleason score, and between correlations are all correlations between cancer samples with different Gleason score. For patients, within correlations are correlations within all cancer samples from the same patient, while between correlations are all correlations between cancer samples from different patients. For the comparison of normal tissue samples to PCa samples in the same versus different patients, within correlations are all correlations between normal samples and cancer samples in the same patients, while between correlations are correlations between all normal samples and cancer samples in different patients.

### Subtraction for normal tissue component

To emphasize the cancer signature in each prostate cancer sample, cancer samples were subtracted for their normal tissue component. This was done by first calculating the average normal signature based on the 40 normal samples with no cancer tissue. Each of 116 cancer samples was then subtracted for their normal component according the content of normal tissue using the following equation:

$$PR_{\mathrm{corrected}}=\mathrm{PR}-\frac{SE_{\mathrm{AVG}}*\mathrm{frac}}{1-\mathrm{frac}}$$

Here *PR* is the original signature for an arbitrary sample, *SE*_*AVG*_ the average normal signature based on all normal samples, and *frac* the fraction of normal tissue in the sample, ranging from 0.1 (very little) to 0.9 (almost entirely consisting of normal tissue). A higher fraction value results in a stronger subtraction of the normal signature. To investigate whether the subtraction also unintentionally emphasized cancer signatures in the normal samples, we subtracted the normal signature from these samples as well using the strongest subtraction factor of 0.9.

### Hierarchical clustering

Hierarchical clustering of the 116 cancer samples was performed using the *Hierarchical clustering (scipy.cluster.hierarchy)* package in python, using *correlation* as distance measure. All other parameters were set to default values. For the clustering, we used GSEA signatures based on the 15 gene sets from Markert et al. after subtraction for the average normal signature. Samples and signatures were sorted according to the *leaves* order from the clustering in both the sample and the signature direction before plotting, so similar samples and signatures are located next to each other. The four new gene sets were subjected to the same GSEA, normalization and normal tissue subtraction as the original 15 gene sets.

## Results

### Validating the four recently published PCa subtypes in fresh frozen radical prostatectomy tissue

Using the 15 gene sets from Markert et al. [3], we used GSEA to calculate a gene set signature for each of our tissue samples (116 PCa and 40 normal). We compared these sample signatures to the four subtypes generated from the cohort subjected to radical prostatectomy from the Memorial Sloan-Kettering Cancer center (Figure 1A) using a statistical test of dependent correlations [38] (See Methods). In Markert et al. [3], two of the subtypes (ESC/PTEN-/Proliferation – BP-E/P/Pr and TMPRSS2-ERGfusion – BP-ERG) were associated with bad prognosis, and another two subtypes (Good Prognosis 1 - GP1 and Good Prognosis 2 - GP2) were associated with good prognosis based on follow up data on survival time. Only few samples (11 PCa and 9 normal at p < 0.05) could be uniquely assigned to one of the four published subtypes (Table 1A). However, a substantial number of the samples (47 PCa and 31 normal, p < 0.05) could be assigned to either the two signatures related to good prognosis or the two signatures related to bad prognosis (Table 1A). The normal tissue samples are exclusively assigned to the subtypes with good prognosis. The PCa samples were mostly assigned to the subtypes with bad prognosis, but a few PCa samples also fell into the subtypes for good prognosis. The general separation of PCa and normal samples into bad and good prognosis categories, respectively, appeared to be robust, where normal samples were assigned to the bad prognosis subtypes only at very high p-value thresholds (p > 0.5) (Figure 1B-Top). Subtype assignments into of samples to good and bad prognosis were also prevalent for samples in the additional validation study, however, in this dataset contamination of cancer signatures in normal samples was also present (Additional file 1: Text S1).

**Table 1:** **Number of samples assigned exclusively and significantly (p < 0.05 and p < 0.25, dependent correlations by Steiger**[38]**) to one of the four PCa subtypes, compared to the number of samples assigned when the four categories are combined into two categories with bad and good prognosis**

**A) Original signature**							
	**p-value threshold**	**BP-E/P/Pr**	**BP-ERG**	**GP1**	**GP2**	**Bad prognosis**	**Good prognosis**
PCa	0.05	10	0	0	1	37	10
	0.25	22	3	2	11	52	21
Normal	0.05	0	0	2	7	0	31
	0.25	0	0	4	17	0	35
**B) Signatures subtracted for the average normal signature**							
	**p-value threshold**	**BP-E/P/Pr**	**BP-ERG**	**GP1**	**GP2**	**Bad prognosis**	**Good prognosis**
PCa	0.05	9	1	0	1	53	4
	0.25	23	5	0	4	73	7
Normal	0.05	0	0	0	6	3	12
	0.25	4	0	2	13	7	19

While only few samples could be assigned exclusively to one of the four subtypes, a substantial number of samples could be assigned to one of the two combined categories of good and bad prognosis.

### Subtype assignment are strongly dependent on sample composition, but independent of Gleason score

PCa tissue samples rarely contain pure cancerous tissue, but usually consist of cancer and normal tissues (stromal and epithelial cells) in various proportions. Thus, PCa samples consisting of 50% cancer and 50% normal cells will have gene expression levels resembling both cancer and normal samples. The tissue composition of each PCa sample is therefore likely to affect the assignments to the two subtype categories of good and bad prognosis. In our fresh frozen radical prostatectomy tissue, a substantial number of tissue samples could not be assigned to the poor or good prognosis categories, even at high p-value thresholds. When investigating the dependency between tissue composition and the number of unassigned samples, we observed a higher number of unassigned cases where samples contained similar proportions of cancer and normal tissue (proportions of 50/50, 60/40 and 40/60) (Figure 1B-Middle). However, the dependency was not absolute, because nine PCa samples with at least 80% cancer tissue, and five normal samples (consisting of 100% benign epithelial and stromal cells) remained unassigned (p < 0.25). Additionally, 10 PCa samples assigned with a good prognosis subtype (p < 0.05) contained high percentages of normal tissue (58% on average, compared to 38% on average for all PCa samples), which may influence the assignment of these samples. When investigating Gleason score dependency on subtyping, we could not identify a preference in sample assignment for any particular Gleason score (Figure 1B-Bottom). Samples with Gleason score 8 or 9 were just as likely to be assigned with poor prognosis as those with Gleason score 6 or 7. For the additional validation data, the effect of normal tissue contamination in cancer samples seemed to have less influence on subtype assignment, and samples with low tumor content, as well as normal samples, could both be assigned to the bad prognoses subtype (Additional file 1: Text S1).

### Subtype assignments are homogenous within samples from the same patient, but not necessarily between patients having the same Gleason score

In our dataset, we had several cancer and normal samples that were extracted from the same patient, giving us the opportunity to investigate the genetic heterogeneity among patients. In cancer samples, we observed high correlations between signatures within each patient (median r = 0.56) compared to correlations between patients (median r = 0.09, p ≈ 0.0, Figure 1C). This is in contrast to the comparison of signature correlations within samples with the same Gleason score (median r = 0.14), and samples with different Gleason scores (median r = 0.08, p = 0.0001). This indicates that signatures retain their within patient homogeneity, despite having different Gleason scores. In other words, two samples with different Gleason score from the same patient are more likely to have correlated gene signatures than two samples with the same Gleason score taken from different patients. In addition, when considering only samples within the same patient, we found no significant increase in the correlation of signatures for samples with the same Gleason score compared to samples with different Gleason score (p = 0.75, Additional file 1: Figure S3).

When comparing similarity using signature correlations of cancer and normal samples in general, normal samples were as expected more similar than cancer samples (median r = 0.10 and 0.55 for cancer and normal respectively, p ≈ 0.0). Furthermore, the signatures for normal and cancer samples taken from the same patient, are more similar compared to signatures from normal and cancer samples taken from different patients (median r = -0.08 and r = -0.27 for same and different patients respectively, p = 0.0006). This observation confirms the dependency of signatures between PCa samples and their surrounding normal tissue [39].

When considering the 21 PCa samples assigned with good prognosis (p < 0.25), 18 of these samples are shared between 12 patients, and only 3 of the other samples from these 12 patients are assigned with poor prognosis. Thus, the good prognosis PCa samples seem to be distributed in a subset of patients with a certain degree of similarity in signatures. This observation supports the hypothesis that the good prognosis PCa samples not only have higher levels of normal tissue, but also represent a less aggressive subtype of PCa. Moreover, PCa samples with Gleason score 6 and 7 taken from patients who also harbored samples with Gleason score 8 and 9, were either unclassified or assigned with poor prognosis, also supporting this hypothesis.

### Subtraction of an average normal signature to emphasize the cancer component in the heterogeneous tissue improves subtype classification of PCa samples

We have shown that the heterogeneous tissue composition of PCa samples affects subtype assignments, especially when the proportions of cancer and normal tissue are similar. To emphasize the molecular cancer component in each sample, we used all normal samples to construct an average normal signature based on the 15 gene sets. The average normal signature was then subtracted from the original signature for each cancer sample, where the proportion of subtraction was relative to the normal tissue component from the histopathological evaluation. This procedure improved the subtype assignment for several cancer samples. Specifically, the number of PCa samples assigned with bad prognosis increased from 37 to 53 (p < 0.05, at p < 0.25 this number increased from 52 to 73, Table 1B). All samples assigned with poor prognosis after subtraction were unassigned before subtraction. All samples initially assigned with poor prognosis retained their assignment after subtraction. As expected, no new samples were assigned with good prognosis due to the signature similarity between cancer samples with good prognosis normal samples.After subtraction of an average normal signature, within patient correlations of signatures improved (median r = 0.70 compared to 0.55). This was also the case for samples within the same Gleason score (median r = 0.37 compared to 0.14) (Figure 1D). To investigate whether the subtraction procedure could over-emphasize cancer signatures in normal samples, we applied the subtraction strategy to the normal samples as well (composition subtraction factor of 0.9, the highest factor used for any sample in the study, see Methods). All samples initially assigned with good prognosis either retained their assignment, or changed to unassigned (p < 0.05). We thus conclude that the subtraction procedure improves the subtype classification of cancer samples, and does not over-emphasize cancer signatures in normal samples.

Subtraction of the same average normal signature in the validation samples also improved subtype classification in the additional validation dataset, however, for this dataset enrichment of cancer signatures for some of the normal samples was also observed. (Additional file 1: Text S1).

### Samples with signatures enriched for ESC, MYC and ERG-fusion are characterized by low Gleason score but potentially poor prognosis

**Figure 2:**
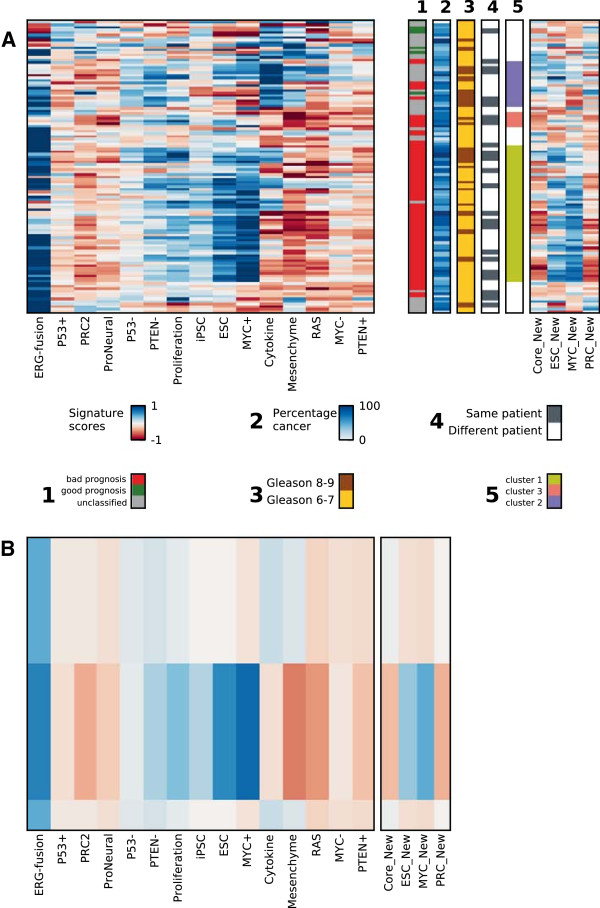
**Hierarchical clustering of signatures for all PCa samples reveal one dominating cluster (cluster 1, green) with low Gleason scores, classified with poor prognosis profiles, and enriched for ERG-fusion, ESC and MYC + gene sets**. Signature scores after subtraction of the average normal signature were used for the clustering. **A)** Heatmap of signatures based on GSEA scores for the 15 gene sets in 116 cancer samples. The additional bars show meta-data for each sample, from left to right: 1) Samples assigned with poor prognosis (red), good prognosis (green) and unclassified (grey) at a p-value threshold of 0.25; 2) Percentage cancer tissue, 3) Gleason score, Gleason 6-7 (yellow), Gleason 8-9 (brown); 4) Whether a sample clusters with an adjacent sample from the same patient, yes (dark blue), no (white),5) Interesting clusters found in the heatmap, see main text for details. Two of the most important gene sets were validated by four related gene sets from recent studies, where the ESC_New, MYC_New and PRC_New gene set values correlated well with the previous ESC, MYC + and PRC2 gene set scores. Note that these four gene sets were not used for the hierarchical clustering, and are thus independent validations. **B)** Average gene set values over all samples in cluster 1, and compared to the average over all other cancer samples, emphasizing the characteristics for cluster 1.

To investigate how our dataset of PCa samples clustered based on signatures subtracted for their normal tissue components, a hierarchical clustering of all subtracted cancer samples was performed and presented as a heatmap in Figure 2A. The overall pattern shows that the samples are fairly heterogeneous with respect to their signatures, but one major cluster (containing 54 of the 116 PCa samples, cluster 1 in Figure 2A) was separated from the other samples by increased values for the ERG-fusion, Embryonic Stem Cell (ESC) and MYC-positive (MYC+) gene sets (average gene set values for this cluster compared to other samples are shown in Figure 2B). Although these three gene sets generally showed increased values in samples from this cluster, the values were not always increased at the same time. Typically, two out of the three gene set values were increased in a single sample. Among these three gene sets, the ESC and MYC + sets correlated with each other (r = 0.64), but neither correlated with the ERG-fusion set (r = -0.1 and r = -0.2 for ESC and MYC + respectively). Additionally, we also observed increased values for the PTEN- and Proliferation gene sets, and depletion of the PRC2, Mesenchyme, RAS and Cytokine gene sets, however, these enrichments and depletions were weaker and less consistent across the samples in cluster 1. All 54 samples except one in the identified cluster were assigned with bad prognosis (p < 0.25). Interestingly, most of these samples (n = 38) have low Gleason scores of 6 and 7, despite being assigned with bad prognosis. Increased values of gene set scores related to bad prognoses subtypes were also observed in a substantial number of samples with Gleason score 6 and 7 in the additional validation dataset (Additional file 1: Text S1). The combination of low Gleason score with bad prognosis may indicate that high gene set values of ERG-fusion, MYC + and ESC serve as early markers for a more dangerous PCa subtype.

To validate the ERG-fusion, MYC + and ESC gene set enrichments in our study, two recently published independent gene sets for ESC and MYC were used (ESC-New [18] and MYC_New [25]). Average GSEA scores for these gene sets from the normal samples were also subtracted for the new gene sets. The study containing the MYC_New gene set also included additional gene sets for Polycomb (PRC_New) and ES core pluripotency factors (Core_New) which were also included in the validation (Figure 2). Good correlations between the new and existing gene set scores over all PCa samples were observed (r = 0.62 and r = 0.88 for ESC/ESC_New and MYC+/MYC_New, respectively), despite poor overlap of genes both from the new and the existing gene sets (only 28 of 189 for ESC and 18 of 355 for MYC). This clearly indicates that GSEA calculated gene set scores are quite robust with respect to the selection of genes in the signature, and highlights the benefit of using gene sets rather than individual genes for molecular characterization of PCa. The PRC_New gene set scores were reduced in the samples from the dominant cluster (cluster 1), which was in accordance with the observed depletion in the existing PRC2 gene set scores (r = 0.75 for PRC2/PRC_New, 196 of 451 genes overlap).

To further validate the significance of the individual gene sets (ERG-fusion, ESC, ESC_New, MYC and MYC_New) in separating PCa samples with poor and good prognosis, we compared the individual gene set scores for these five signatures between the following sample sets: i) The 54 cancer samples with bad prognosis from cluster 1 and highlighted in Figure 2, ii) The 21 cancer samples initially assigned with good prognosis (p < 0.25, Table 1A) and iii) The 40 normal samples. The ESC, MYC + and MYC_New show strongly elevated gene set scores over the 54 samples with bad prognosis, compared to the good prognosis and normal samples (Figure 1E), while the elevation of ESC_New and ERG_fusion gene set scores are less pronounced.

In addition to the dominant cluster 1, two smaller clusters (2; purple, and 3; pink, Figure 2) with samples sharing elevated scores of certain gene sets were also observed. One of these clusters (cluster 2) contained samples particularly enriched for the Cytokine gene set. The majority of samples (12 out of 18) in this cluster where categorized with either Gleason 8 or 9, and the Cytokine gene set generally showed increased values in samples with higher Gleason scores (Additional file 1: Figure S4). The other small cluster (cluster 3) contained samples showing specific depletion in the Mesenchyme and ProNeural gene set scores. Most samples in this cluster (4 out of 6) were from the same patient, indicating that this patient may have a less common type of PCa. The general similarity in signatures among PCa samples from the same patient was also evident in the hierarchical clusters, where 45 samples shared a cluster neighbor with a sample from the same patient (only 5 samples would be expected to have this property by chance). Finally, the low influence of tissue composition on the sample clustering underlines the effect of the subtraction procedure in limiting the tissue heterogeneity effect.

## Discussion

Detection of PCa molecular subtypes revealing the prognosis at an early stage of cancer development would be helpful in stratifying patients to personalized treatment. New prognostic models independent of Gleason grading is the ultimate goal for adding new prognostic tools into the clinic. In this study, we used fresh frozen prostatectomy tissue of high molecular quality [17] thoroughly evaluated by histopathology to validate previous and assess new molecular subtypes using microarray gene technology.

The histopathological evaluation of tissue composition (amount of cancer and normal benign structures of stromal and epithelial cells) in our fresh frozen sample material enabled us to investigate the effect of sample composition on PCa subtyping. By subtraction of an average normal signature, we achieved an improved classification of PCa samples. Still, a substantial number of samples could not be assigned to any of the categories. This could be due to samples containing subtype characteristics not captured by the gene sets used here, or that an even more specific subtraction method has to be used. However, the results clearly underline the importance of accounting for tissue heterogeneity in the analyses. Important information may be hidden and lost because the variation due to tissue composition heterogeneity overshadows the biological variation of interest, and this is also valid for analyses of other molecular properties than the transcriptome. The identification of PCa samples with good prognosis signatures are particularly challenging due to the molecular similarities between these cancer subtypes and normal samples. A consequence of this may be that cancers with poor prognosis characteristics are assigned with good prognosis because of high normal tissue content in the sample.

PCa often has a multi-nodal character, being present at separated and presumably independent foci in the prostate. Some studies suggest that multifocal PCa most likely has a monoclonal origin [40], making within patient variation between PCa samples less than the inter-patient variation, even when samples are taken from different foci. In our study, with many samples taken from the same patient, we observe considerably higher signature variability between than within patients, even when the samples from the same patient have different Gleason scores. This supports the hypothesis of a monoclonal origin, but is in contrast to a previous study targeting multi-nodality where the presence of i.e. gene fusions was reported to vary in different foci. However, or study was designed to obtain tissue of different Gleason grades, and no specific focus was put on multi-nodality. In this setting, samples taken from different foci in each patient consistently showed similarities.

When subtracting the average normal signature from the PCa samples, hierarchical clustering revealed a particular dominant cluster representing enrichments in the ERG-fusion, ESC and MYC + gene sets. In our data, the scores for these three gene sets were markedly higher in samples of poor prognosis compared cancers with good prognosis and normal samples, which should make them able to discriminate between the categories. Additionally, the gene set scores for ESC and MYC + were validated by recent gene sets for ESC and MYC from independent studies. Genes from the additional ESC and MYC gene-sets overlapped poorly with genes from the ESC and MYC + gene-sets in Markert et al. [3], however the gene set scores were highly correlated. This corroborates the robustness of using gene sets related to specific properties or cell functions rather than individual genes for classification and characterization. A substantial number of PCa samples (38 of 116) assigned with poor clinical prognosis had a low Gleason score (6 or 7) and consistently showed high scores for these three gene sets. This indicates that the features of these cancer samples are quite common in PCa. Additionally, there were consistent signatures from samples taken from the same patient independent of different Gleason scores. ERG-fusion, ESC and MYC + gene sets are thus interesting due to the robustness to sample variability within the same patient, their evidence at early stage PCa and the ability to predict clinical poor prognosis.

In Markert et al. [3], there was a special focus on the bad prognostic signature enriched for the ESC, PTEN- and P53- signatures, while enrichment for ERG-fusion was characteristic for an intermediate prognosis. Though we observe some enrichment of the PTEN- gene set, we find that both the PTEN- and the P53- gene sets are more enriched in samples with Gleason score 8 and 9, indicating that they are later events in PCa progression [28]. Similar to Markert et al. [3], we also observe an enrichment of the Mesenchyme and Cytokine gene sets in normal samples and cancers with good prognosis. However, these gene sets are also enriched in cancers with Gleason score 8 and 9, thus making them too ambiguous for early stage PCa assessment (Additional file 1: Figure S4). It should also be noted that the P53- gene set only contain four genes, of which only two are present in our micro-array data, so scores for this gene set may lack robustness.

There are two important limitations in our study which need to be mentioned. First, our data currently lack follow-up information with respect to recurrence and patient survival. This means that the assessment of good and poor prognosis in our study relies on follow-up data on patient survival time from Markert et al. [3]. Follow up information from the additional validation dataset showed recurrence in 13 of 29 patients assigned with a bad prognosis signature together with a Gleason score of 6 or 7, however, these numbers could not be compared to recurrence measures for samples assigned with good prognosis due to insufficient data (Additional file 1: Text S1). However, for early localized PCa with low Gleason scores, follow up data may be of limited value, since a successful radical prostatectomy likely prevents recurrence, regardless of whether the removed tumor contained a potentially dangerous subtype or not. Nevertheless, the most important poor prognosis signature characteristics were clearly reproducible in the signatures calculated on low Gleason samples in our data, as well as on the validation data, and are thus used as an indication of potentially dangerous molecular characteristics which can be identified at an early stage. To fully answer the question of whether these tumors are more likely to become lethal if they remain in the patient, tissue biopsies sampled from tumors over time is necessary, but such samples are currently not readily available. In addition, our study adds value by analyzing the signatures in the context of tissue heterogeneity and intra-patient variability, both important in a clinical perspective. Second, prostate tumors classified with Gleason score 6 are generally not surgically removed, so samples with Gleason score 6 are all taken from PCa tissue which also harbored tumors with higher Gleason score (most often a Gleason score of 7). We generally assume that poor prognosis signatures in these samples are indicative of an “early version” of the tumor with higher Gleason. However, we also acknowledge the possibility that the Gleason 6 samples may be exposed to a field effect from the higher Gleason tumor, resulting in signatures resembling poorer prognosis. However, the prevalent presence of poor prognosis signatures in samples from Gleason 6 patients in the additional validation set argues against this.

## Conclusions

We have validated gene expression signatures used for identification of prognostic PCa subtypes in a new patient cohort from standardized tissue material of high molecular quality. Our data provide new information on a subtype of cancer samples enriched for gene sets related to ERG-fusion, ESC and MYC + indicating poor prognosis on an early stage of PCa development (low Gleason score). The identified signatures seem to be common for PCa, consistent within the same patient and have the potential to give important information on dangerous PCa beyond Gleason grading. These PCa subtypes are promising in a clinical setting for treatment planning and as inclusion criteria for active surveillance programs. For the future, we need to validate the signatures further in larger patient cohorts and develop more sophisticated regimes to correct for tissue composition heterogeneity.

## Abbreviations

GSEA: Gene set enrichment analysis; PCa: Prostate cancer.

## Competing interests

The authors declare that they have no competing interests.

## Authors’ contributions

HB and MBT provided data. MBR, HB and MBT designed the analysis. MBR performed the analysis. MBR, HB, MBT, FD, AA and TFB wrote the manuscript. All authors read and approved the final manuscript.

## Pre-publication history

The pre-publication history for this paper can be accessed here:

http://www.biomedcentral.com/1755-8794/7/50/prepub

## Supplementary Material

Additional file 1 Supplementary tables, text and figures S1 – S4. Click here for file

Additional file 2 Sample composition and patient number for each sample. Click here for file

Additional file 3 Genes and gene sets. Click here for file
