# Supplementary material for: Gene signatures ESC, MYC and ERG-fusion are early markers of a potentially dangerous subtype of prostate cancer
Source: BMC Med Genomics. 2014 Aug 12;7:50. doi: 10.1186/1755-8794-7-50 (PMC4147934; doi:10.1186/1755-8794-7-50)

Additional File 1

Supplementary overview and captions

Supplementary Text S1

Results from additional validation in an independent microarray dataset (GEO accession GSE8218)

Supplementary Table S2

Genes present in our microarray data compared to the total number of genes in 21 PCa related gene sets from Markert et al., and four additional gene sets downloaded from independent studies. GSEA scores for cytokine, ERG-fusion, ESC and iPSC gene sets were combined, resulting in 15 gene sets used for analysis.

Supplementary Figure S3

Signatures are independent of Gleason score within samples taken from the same patient. Signature correlations between samples from a patient with the same Gleason score are not significantly higher than signature correlations between samples from a patient with different Gleason score.

Supplementary Figure S4

Gene sets enriched in samples with high Gleason scores. The Cytokine and Mesenchyme gene sets are enriched in normal samples (in addition to samples with Gleason score of 6 or 7 assigned with good prognoses). However, the enrichment of these signatures in samples with higher Gleason scores of 8 and 9 makes them too ambiguous markers. The P53- and PTEN-gene set scores are correlated with Gleason score, and are especially prevalent in samples with Gleason scores of 8 and 9.

Supplementary Text S1 – Validation using dataset GSE8218

To validate the findings in our own dataset with independent data, we selected a publicly available microarray datasets (GEO accession GSE8218 [1, 2]), which included detailed information on sample composition, Gleason score and time of tumor recurrence after surgery. To assess the quality of the data, we calculated the average GSEA score profile correlation over all samples between the four related ERG-fusion signatures, the two ESC-signatures and the two MYC-signatures, respectively, assuming they would produce correlated score profiles if the data were of good quality. This was the case both for our own data (average cc=0.95) and the GSE8218 data (average cc=0.89). We thus concluded that this dataset would be appropriate to validate results from our own dataset.

The microarray data contained in GSE8218 consist of 148 PCa tissue samples, where 136 samples contain detailed information on sample composition. For the validation we only used the samples with sample composition information. Each of the 136 samples contained percentage assessment of the four tissue types, cancer, stroma, benign epithel and atrophic glands. Because our data did not distinguish between atrophic glands and benign epithel, the percentages of these two tissue types were added together in the further analysis. We defined normal samples as those with a cancer content of zero percent, which divided the dataset into 71 normal and 65 PCa samples. Thus there are a higher number of normal samples in the validation datasets compared to our own data. The validation data also differed from our data with respect to other properties: PCa tissue samples in the validation data had a higher average stroma content (56 % on average compared to 35% for our data), were analysed using microarrays from a different vendor (Affymetrix U133A compared to Illumina Human HT-12), were characterized with Gleason scores at the patient level (compared to sample level in our data) and were harvested using a different procedure. Although we could not find an exact protocol for harvesting in the references for the validation data, the normal samples seems to be generally taken closer to the tumor compared with normal samples in our data [2]. Overall, the validation datasets contained expression measurements for 22 283 probes representing 12 495 unique genes.

Gene Set Enrichments Analysis (GSEA) followed by subtype assignment using the statistical test of dependent correlations resulted in subtype assignments for a substantial number of samples from the validation data. Interestingly, the subtype assignments were highly biased towards the BP-E/P/Pr subtype related to bad prognosis and the GP1 subtype related to good prognosis (Table S1.1A). This is somewhat in contrast to assignments on our own samples, where only few samples had confident assignments to any one of the four initial subtypes. Very few of the validation samples could be assigned exclusively to the BP-ERG or GP2 subtypes, which is in concordance with assignments on our data. Subtype assignments improved in both datasets when the four initial subtypes were merged into two subtypes of bad and good prognosis. Most of the normal samples in the validation set were assigned with good prognosis, however, in contrast to assignments on our samples, we also observed normal samples with signatures characteristic for bad prognosis. A few cancer samples were also assigned with good prognosis, as was also the case in our data.

Subtype assignments improved when the normal tissue component was subtracted from each sample (Table S1.1B). For this we used the average normal signature calculated from the 40 normal samples in our own data. The improved assignments for the validation data thus show that our normal tissue signature may have applicability to other datasets were the sample composition is known. A possible disadvantage is that the subtraction may also emphasize

cancer signatures with bad prognosis in normal tissue, which seems to be the case in some of the validation samples. However, it is difficult to conclude whether these signatures are artefacts, or actually represent an identification of true cancer characteristics in normal samples. In the validation data we observe that several of the normal samples were assigned to the bad prognosis subtype, even without subtraction of the normal tissue component, which may indicate that cancer characteristics are present in the normal tissue in these samples. The presence of cancer signatures in normal tissue probably explains why subtype assignments for the validation data are less dependent on sample composition compared to our own data (Figure S1.1). We suggest that the observed differences can be attributed to variations in harvesting procedures for the two studies. In our study, normal samples were harvested as distant as possible from the tumor on the frozen tissue slice, to limit cancer contamination to the normal tissue. Thus these normal samples consistently lack cancer characteristics, even after subtraction of the benign tissue component. These observations highlight the fact that harvesting procedures should be taken into account when comparing datasets, adding further to the challenge in inter-study comparisons using PCa tissue from patient cohorts.

Table S1.1 : Subtype assignments of validation data from GEO accession GSE8218 for A) uncorrected sample signatures, and B) sample signatures subtracted for their normal tissue component.

A)

	p-value threshold	BP-E/P/Pr	BP-ERG	GP1	GP2	Bad prognosis	Good prognosis
PCa	0.05	13	1	8	0	31	11
	0.25	24	4	11	1	39	14
Normal	0.05	5	0	12	3	6	37
	0.25	8	0	21	12	9	49

B)

	p-value threshold	BP-E/P/Pr	BP-ERG	GP1	GP2	Bad prognosis	Good prognosis
PCa	0.05	14	1	4	0	43	4
	0.25	26	2	7	0	48	7
Normal	0.05	7	0	9	0	16	14
	0.25	16	2	15	1	23	22

The 148 samples in GSE8218 were taken from 78 patients with information on Gleason score and time of tumor recurrence after surgery. Of these, only 51 patients (57 samples) had an associated sample with tumor content higher than 10% (In our data all PCa samples had a tumor content of at least 10%). Further stratification of these samples revealed nine samples (from nine patients) with high Gleason >= 8, 31 samples (from 29 patients) with low Gleason <= 7 assigned with bad prognosis, and eight samples (from eight patients) with low Gleason <= 7 assigned with good prognosis (p-value < 0.25). We were thus able to identify bad prognosis signatures in a substantial number of samples with Gleason score 6 and 7, thus confirming observations in our own data. These samples were generally characterized with enrichment for combinations of ERG-fusion, ESC and MYC+ gene set scores (Figure S1.2). We were not able to assess the enrichment of these gene-sets in the good prognosis category, due to various insufficiencies in these samples (see below). In addition we confirmed patterns observed in both our data and in data used by Markert et al. [3], linking loss of PTEN and P53

to samples with high Gleason (Figure S1.2). However, these lesions are also occasionally found in samples with lower Gleason.

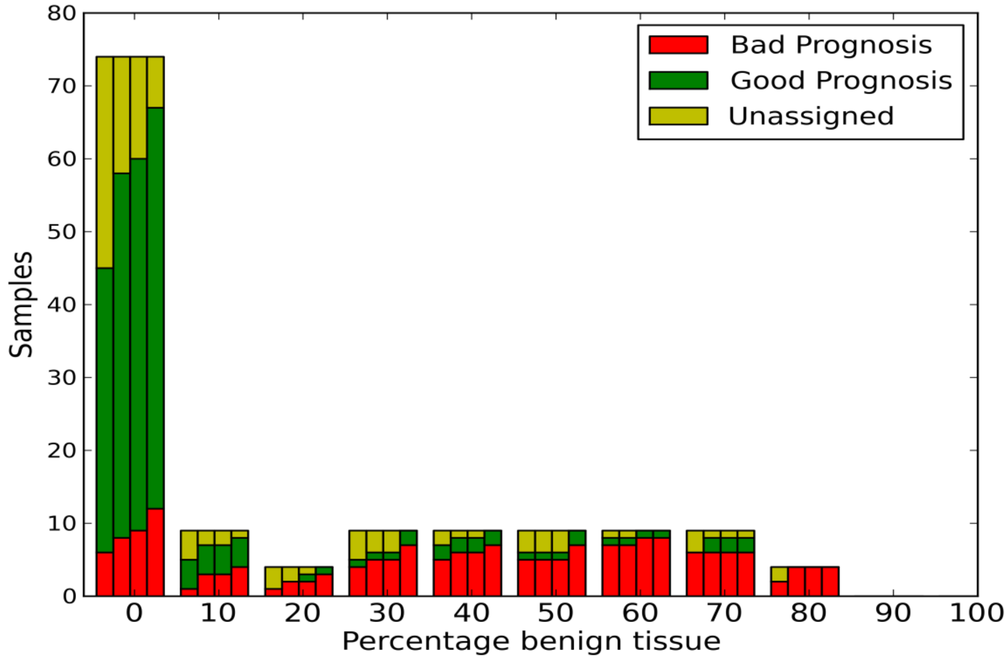

Figure S1.1: Subtype assignment dependence on sample composition. Of the cancer samples assigned with good prognosis, many contain a high percentage of normal tissue (90%). Normal tissue in the validation also displays cancer characteristics, evident by the normal samples assigned with bad prognosis. P-values from left to right: 0.05,0.15,0.25,0.50.

In the validation data we found Gleason score to be the best marker for recurrence, with all nine samples with Gleason score >= 8 showing fast (within 5 months) or intermediate (within 12 months) recurrence. Of the patients assigned with low Gleason scores together with bad prognosis signatures, 13 patients (13 samples) showed recurrence, and 16 patients (18 samples) showed no recurrence. This is expected, since tumors with low Gleason scores mostly represent localized PCa. In most of these cases successful surgery will lead to survival, regardless of whether the tumor is likely to progress into a lethal subtype or not. The signatures and gene-sets presented here were previously associated with recurrence and survival by Markert et al. [3] using samples with both high and low Gleason scores. The interesting observation in our study is that certain gene-sets from these signatures are unrelated to Gleason score, and generally enriched in samples with low Gleason, indicating their potential as early markers. Of these gene-sets, particularly ERG-fusion, ESC and MYC+ show this property. The gene-sets with best relation to recurrence were, interestingly, reduced enrichment of PRC2 as well as loss of P53 (Figure S1.2). The P53- feature was also highlighted in Markert et al. [3]. The recurrence trends observed for these gene-sets for the validation data are only subtle, and not statistically significant at a p-value cutoff of 0.05 (p-values 0.16 and 0.30 for PRC2 and P53-, respectively).

One observation which would strengthen the importance of the bad prognosis signatures would be the lack of recurrence in samples assigned with good prognosis. However, data from the validation set was inconclusive in this respect. Of the eight samples assigned with good prognosis, 4 showed no recurrence, one showed very late recurrence (55 months) and the last three showed faster recurrence. However, two of the samples showing faster recurrence were

both taken from patients having another sample assigned with a poor prognosis subtype. In addition, all samples assigned with good prognosis contained high amounts of normal tissue (>50%), increasing the possibility of tissue confounding.

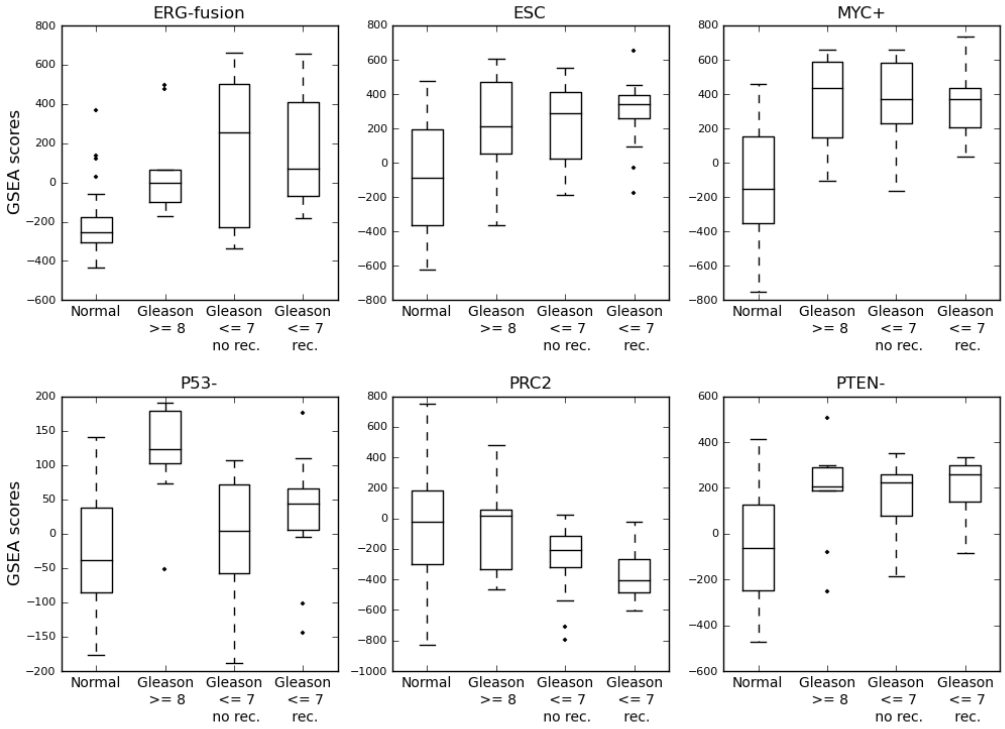

Figure S1.2: Gene-set scores for six gene-sets in four sample groups: Normal samples (71), PCa samples with high Gleason score >= 8 (9 samples), PCa samples with low Gleason score <=7 and no recurrence (18 samples) and PCa samples with low Gleason score <= 7 and recurrence (13 samples).

To conclude, we were able to identify signatures previously associated with poor prognosis in samples with Gleason score 6 and 7 in both our own dataset and the validation dataset, which should facilitate their usefulness as markers for potential early PCa subtype assignment and warrant further studies. The signatures showed stability across two studies with differences in harvesting method, average dataset sample composition and microarray vendor. Assessment of signature performance in low Gleason samples with respect to recurrence was inconclusive.

1. Chen X, Xu S, McClelland M, Rahmatpanah F, Sawyers A, Jia Z, Mercola D: **An accurate prostate cancer prognosticator using a seven-gene signature plus Gleason score and taking cell type heterogeneity into account.** *PLoS One* 2012, **7:**e45178.
2. Wang Y, Xia XQ, Jia Z, Sawyers A, Yao H, Wang-Rodriquez J, Mercola D, McClelland M: **In silico estimates of tissue components in surgical samples based on expression profiling data.** *Cancer Res* 2010, **70:**6448-6455.
3. Markert EK, Mizuno H, Vazquez A, Levine AJ: **Molecular classification of prostate cancer using curated expression signatures.** *Proc Natl Acad Sci U S A* 2011, **108:**21276-21281.

Supplementary Table S2

Gene sets from Markert et al.	Genes in gene set	Genes in microarray data
CYTO1<br>CYTO2	237<br>412	153<br>279
ERG1<br>ERG2<br>ERG3<br>ERGcons	54<br>67<br>49<br>27	42<br>59<br>47<br>27
ESC1<br>ESC2	1076<br>380	715<br>264
MYC+	130	120
MYC-	31	28
Mesenchyme	141	119
P53+	18	15
P53-	4	2
PRC2	654	383
PTEN+	72	54
PTEN-	113	84
Proneural	242	176
Proliferation	183	156
RAS	248	163
iPSC1<br>iPSC2	118<br>340	100<br>290
Additional signatures		
ESC_New	189	171
MYC_New	355	315
PRC_New	451	316
Core_New	75	65

Supplementary Figure S3

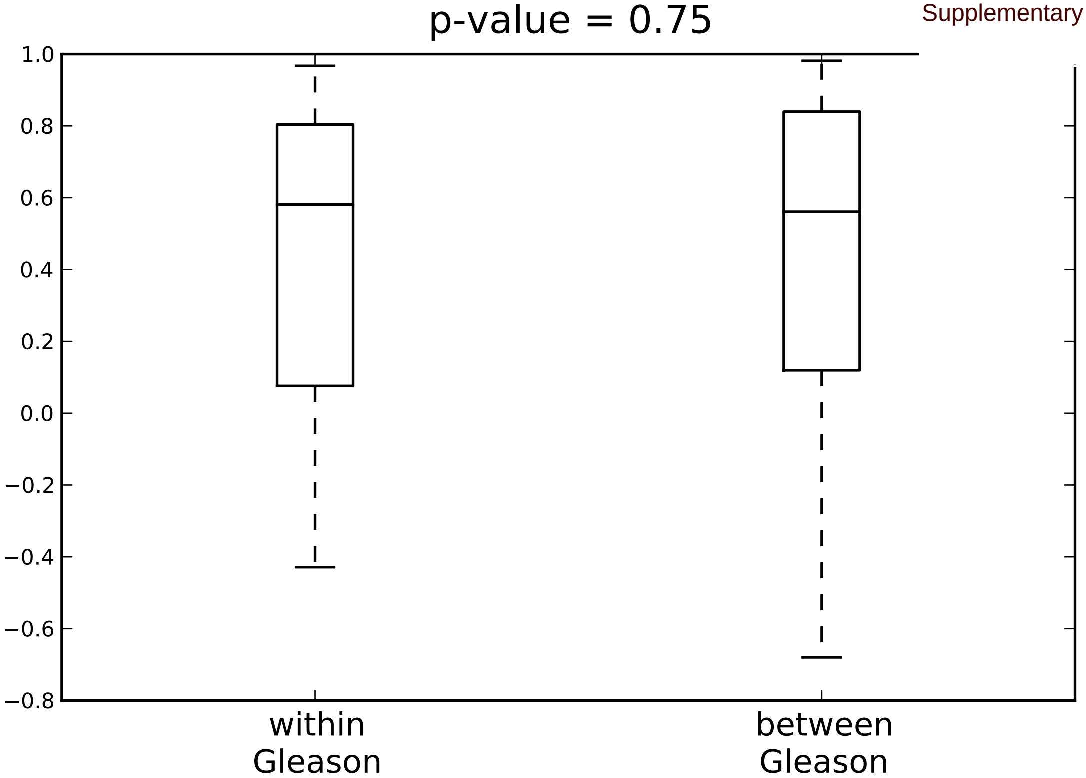

Supplementary Figure S4

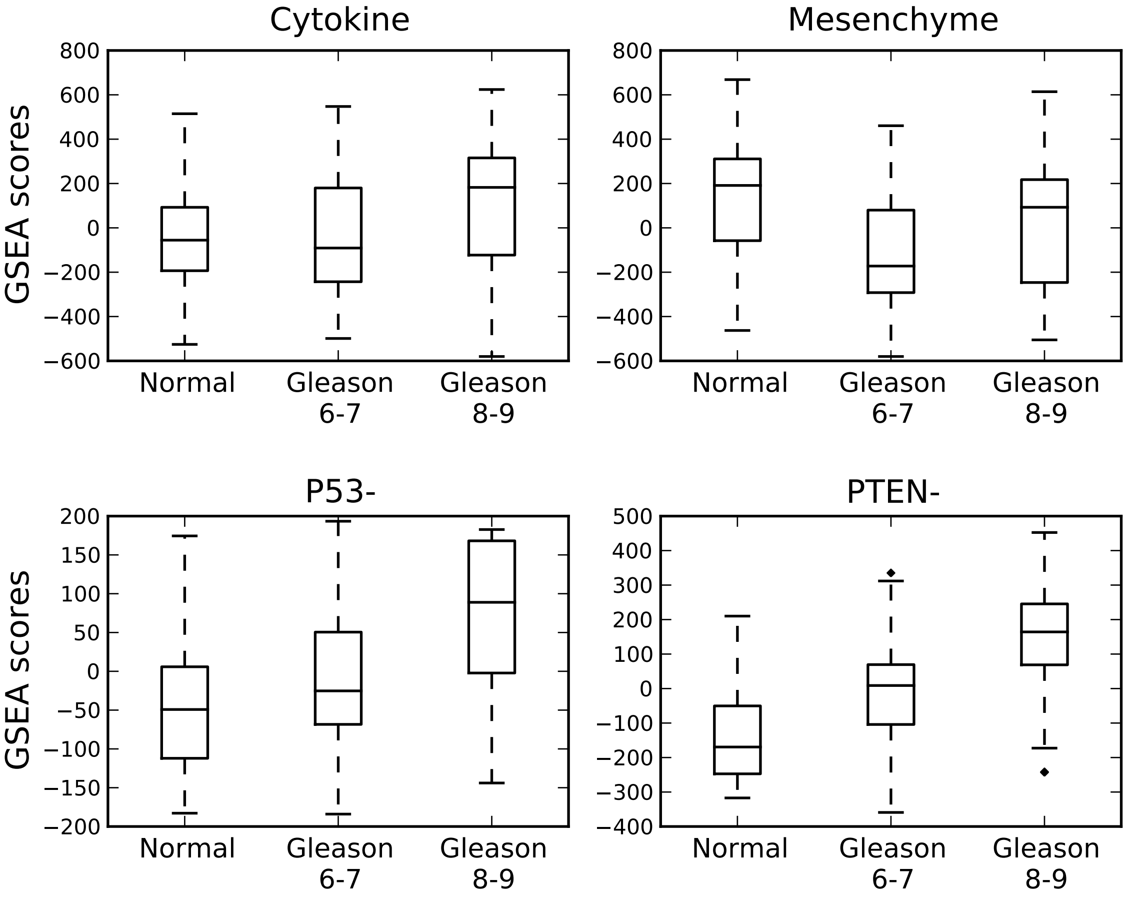

Supplement: Additional file 1 — Supplementary tables, text and figures S1 – S4. [file 1755-8794-7-50-S1.pdf]
